# Too Massive and Too Atypical to Be Benign: The Unexpected Face of a Giant Adrenal Myelolipoma

**DOI:** 10.7759/cureus.101961

**Published:** 2026-01-21

**Authors:** Ijdda Sara, Boukhalfa Ahmed, Ghizlane Elmghari, Nawal El Ansari

**Affiliations:** 1 Department of Endocrinology, Diabetology, Metabolic Diseases and Nutrition, Mohammed VI University Hospital, Marrakech, MAR; 2 Biosciences and Health Research Laboratory/Faculty of Medicine and Pharmacy of Marrakech, Cadi Ayad University, Marrakech, MAR; 3 Faculty of Medicine and Pharmacy of Marrakech, Arrazi Hospital, Mohammed VI University Hospital Center, Marrakech, MAR; 4 Department of Endocrinology and Metabolic Diseases, Arrazi Hospital, Mohammed VI University Hospital Center, Marrakech, MAR; 5 Department of Endocrinology, CHU (Centre Hospitalo-Universitaire) Mohammed VI, Marrakech, MAR

**Keywords:** adrenal gland, adrenal myelolipoma, case report, giant adrenal mass, tumor

## Abstract

Adrenal myelolipomas (AMLs) are rare benign tumors composed of adipose and myeloid tissue. They are most often discovered incidentally and generally remain small in size. However, giant forms can radiologically mimic a malignant tumor, leading to aggressive investigations and treatment. We report the case of a patient who was hospitalized for investigation of a right adrenal mass, revealed by lumbar pain. An abdominal CT scan showed a large, heterodense interhepatorenal mass exerting a mass effect on the ipsilateral kidney with peripheral calcifications. The mass was excised and submitted for histological evaluation. The results of the histological study revealed an AML. Giant myelolipomas are rare and represent a real diagnostic challenge. Their large size, heterogeneous components, and necrotic or hemorrhagic changes can mimic a malignant tumor. Surgery remains indicated in cases of diagnostic uncertainty, compressive symptoms, or tumors larger than 6 cm. Histology remains the only way to establish a definitive diagnosis. This case highlights the difficulty of distinguishing a giant myelolipoma from a malignant adrenal tumor.

## Introduction

Benign adrenal tumours are increasingly being detected thanks to improvements in modern imaging techniques. Among these, adrenal myelolipomas (AMLs) remain an exceptional entity, representing a variant of a mesenchymal tumor composed of mature adipose tissue and myeloid tissue with a variable number of hematopoietic elements, accounting for 3.3 to 6.5% of all adrenal masses [1].

Although generally asymptomatic and small in size, this type of lesion can, in rare cases, reach considerable dimensions, posing a major diagnostic challenge. Indeed, the radiological presentation of a giant AML can be confused with malignant tumors, such as adrenocortical carcinomas or certain retroperitoneal sarcomas, due to its massive volume, slow but progressive growth, and sometimes the heterogeneity of its tissue composition [2]. 

We report here the case of a large AML mistakenly suspected of being a malignant tumor, in order to highlight the diagnostic difficulties, illustrate the radiological and anatomopathological features of this rare tumor, and recall the key elements that can help avoid inappropriate management. This case highlights the importance of rigorous multimodal assessment and accurate clinical-radiological correlation in order to establish a reliable diagnosis and guide optimal management.

## Case presentation

A 53-year-old man, with a history of iron deficiency anemia developing one year earlier under replacement therapy, without etiological investigation, no history of diabetes or hypertension, was admitted for investigation of a right adrenal mass, revealed by right lumbar pain with asthenia. On examination, the patient was in good general health, with no history of excess weight.

**Figure 1:**
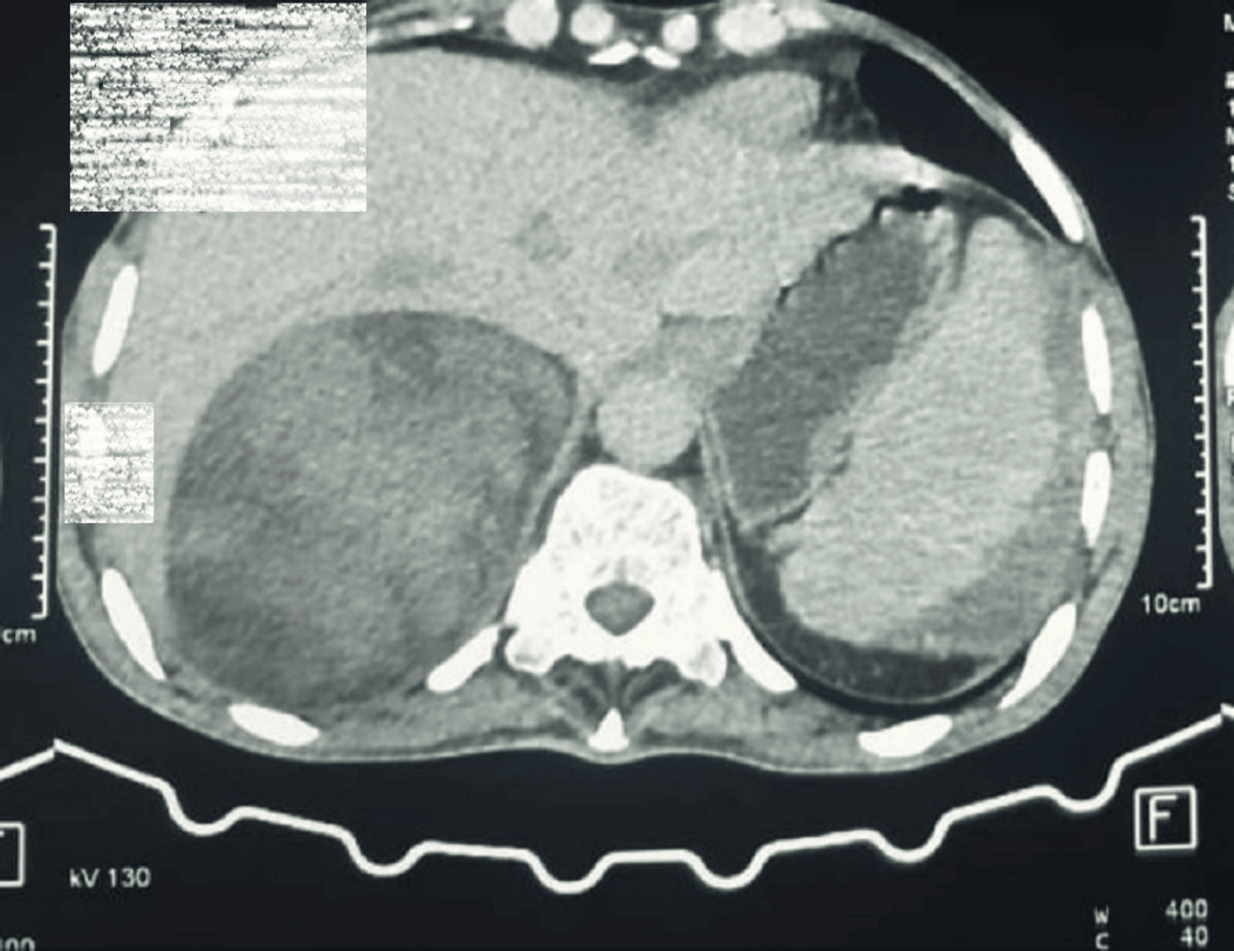
Scannographic image of a large right interhepatic-renal mass

Blood pressure was 110/60 mmHg. Abdominal ultrasound showed a hyperechoic tissue mass in the right adrenal gland, confirmed by an abdominal CT scan, which revealed a rounded, well-defined interhepatorenal mass heterodense with a hypodense component (-11UH) and a tissue component (30UH), slightly enhanced after contrast agent injection, measuring 9.4*11.2*12.6 cm, initially suggesting an adrenal origin. It comes into contact with the lower surface of the liver without any detectable fatty border. At the bottom, it exerts a mass effect on the ipsilateral kidney, with moderate effusion and peripheral calcifications (Figure 1).

Biological assessments for hormonal hypersecretion, particularly free urinary cortisol, a low-dose dexamethasone suppression test and methoxylated derivatives, were normal, while CRP was around 167 mg/l with no notable focus of infection (Table 1). 

**Table 1:** Hormonal and biochemical assessment of our patient

		Normal range	Patient's results
24-hour urinary free cortisol		4.3-176 μg/24 hours	58 ug/24 hours
Morning cortisol after 1 mg overnight dexamethasone		<1.8 μg/dL	1.7 μg/dL
24-hour urinary metanephrines and normetanephrines	Metanephrine	0.3-1.2 μmol/24 hours	0.84 μmol/24 hours
	Normetanephrine	0.8-3 μmol/24 hours	1.50 μmol/24 hours
	3-methoxytyramine	0.1-0.4 μmol/24 hours	0.96 μmol/24 hours
CRP		<5mg/l	167 mg/l

**Figure 2:**
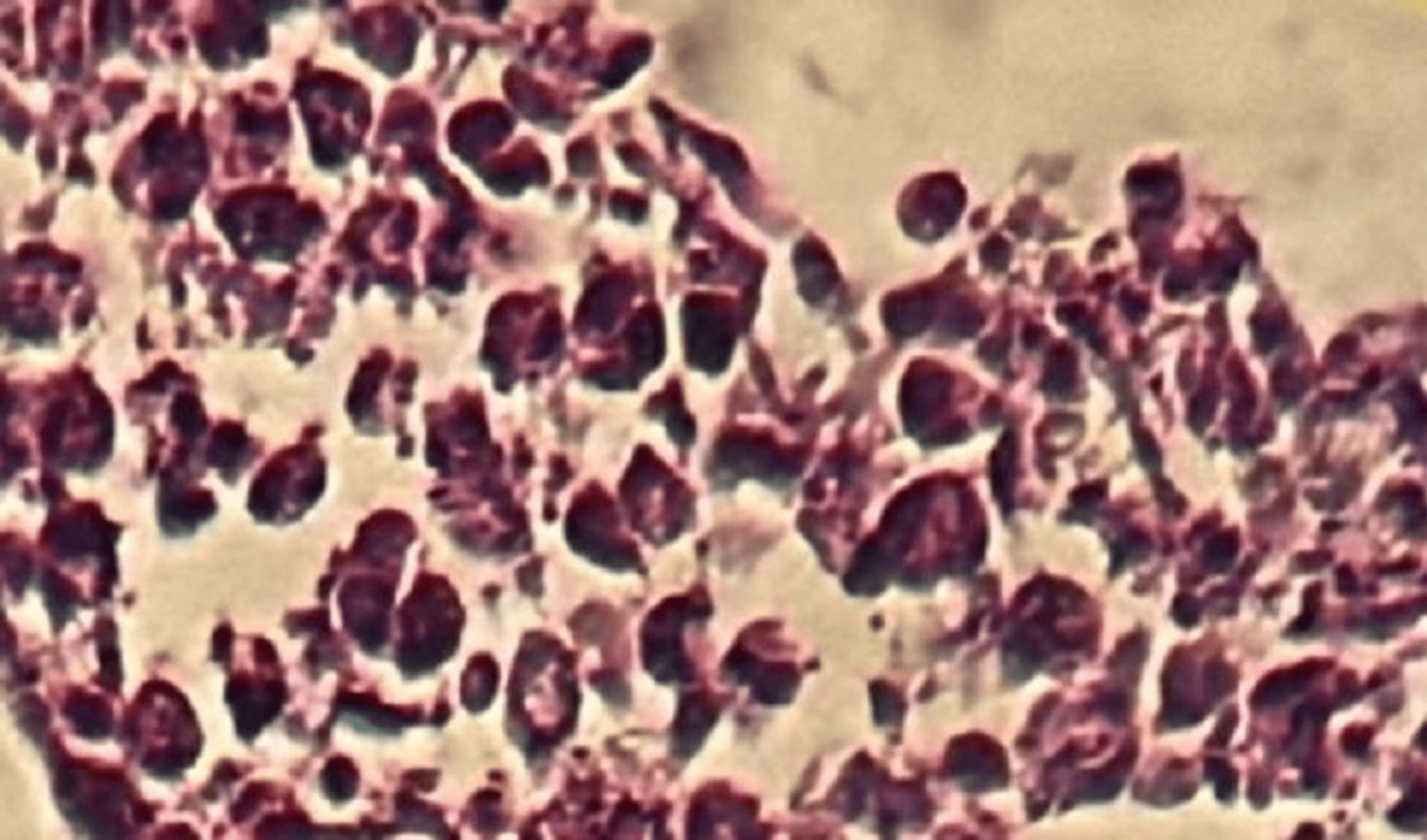
Round cell proliferation (HE X 40)

**Figure 3:**
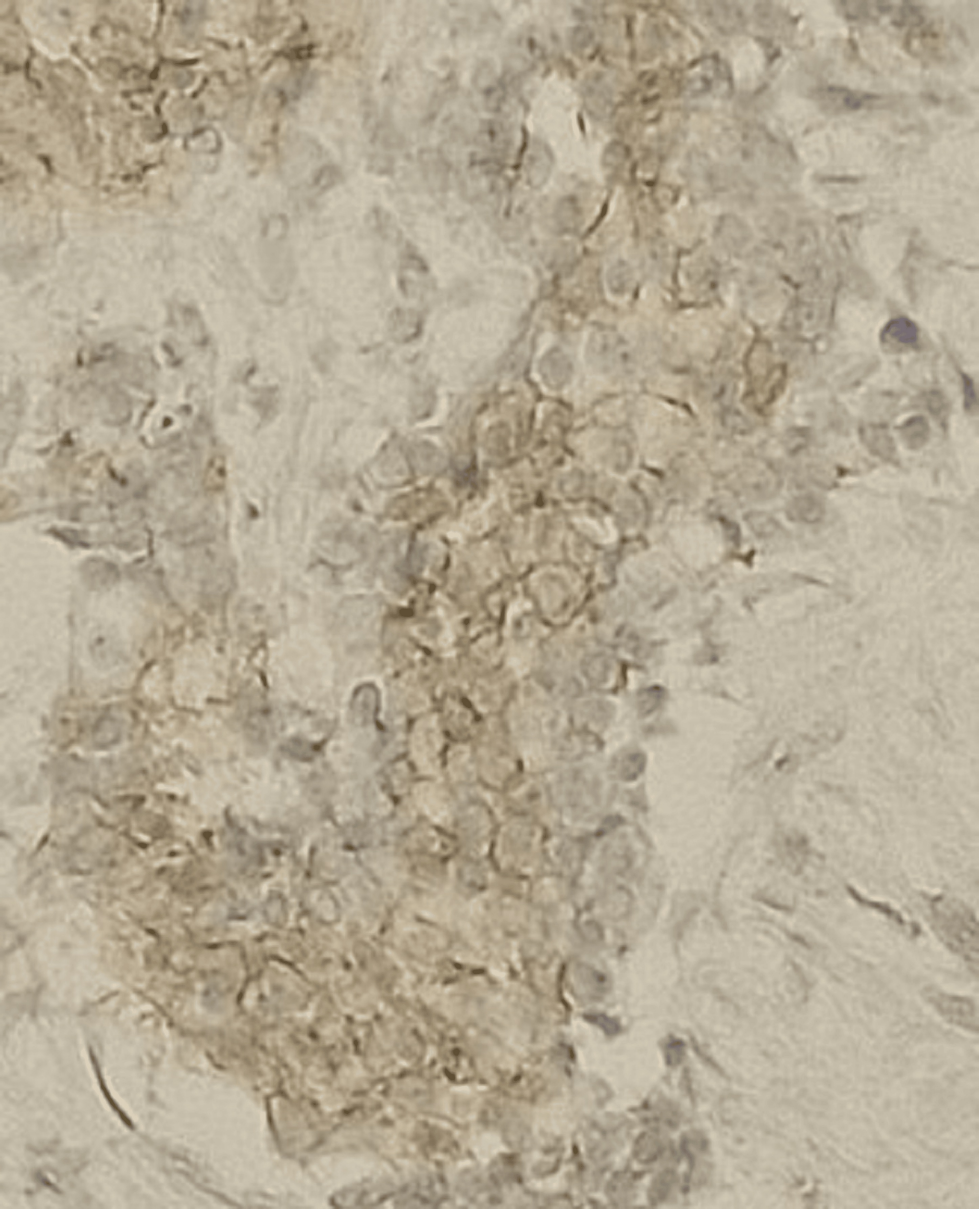
Membrane labeling of round cells by anti-CD99 antibody, suggestive of Ewing's sarcoma

The patient underwent an ultrasound-guided biopsy, with immunohistochemical analysis suggesting Ewing's sarcoma (Figure 2 and Figure 3). 

**Figure 4:**
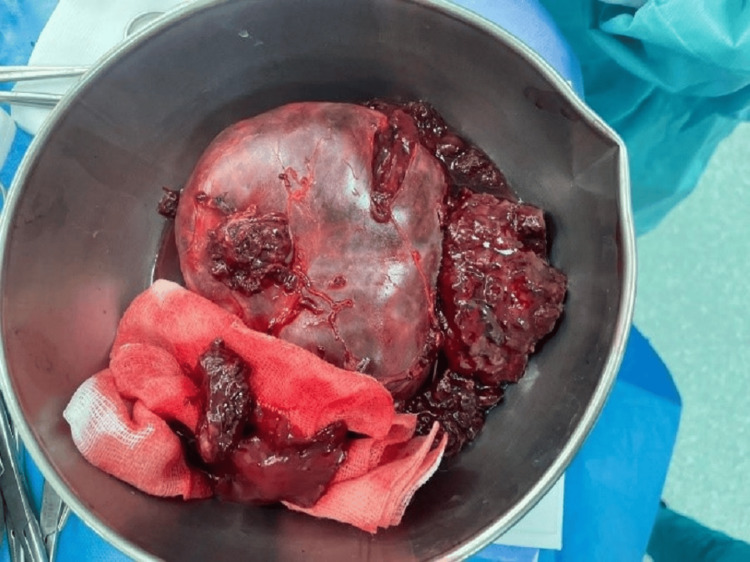
Postoperative image of the resected adrenal mass

The patient then underwent a tumorectomy, with intraoperative exploration revealing a large mass measuring approximately 15 cm between the liver and kidney, infiltrating the right adrenal gland (Figure 4). The postoperative care was uneventful.

**Figure 5:**
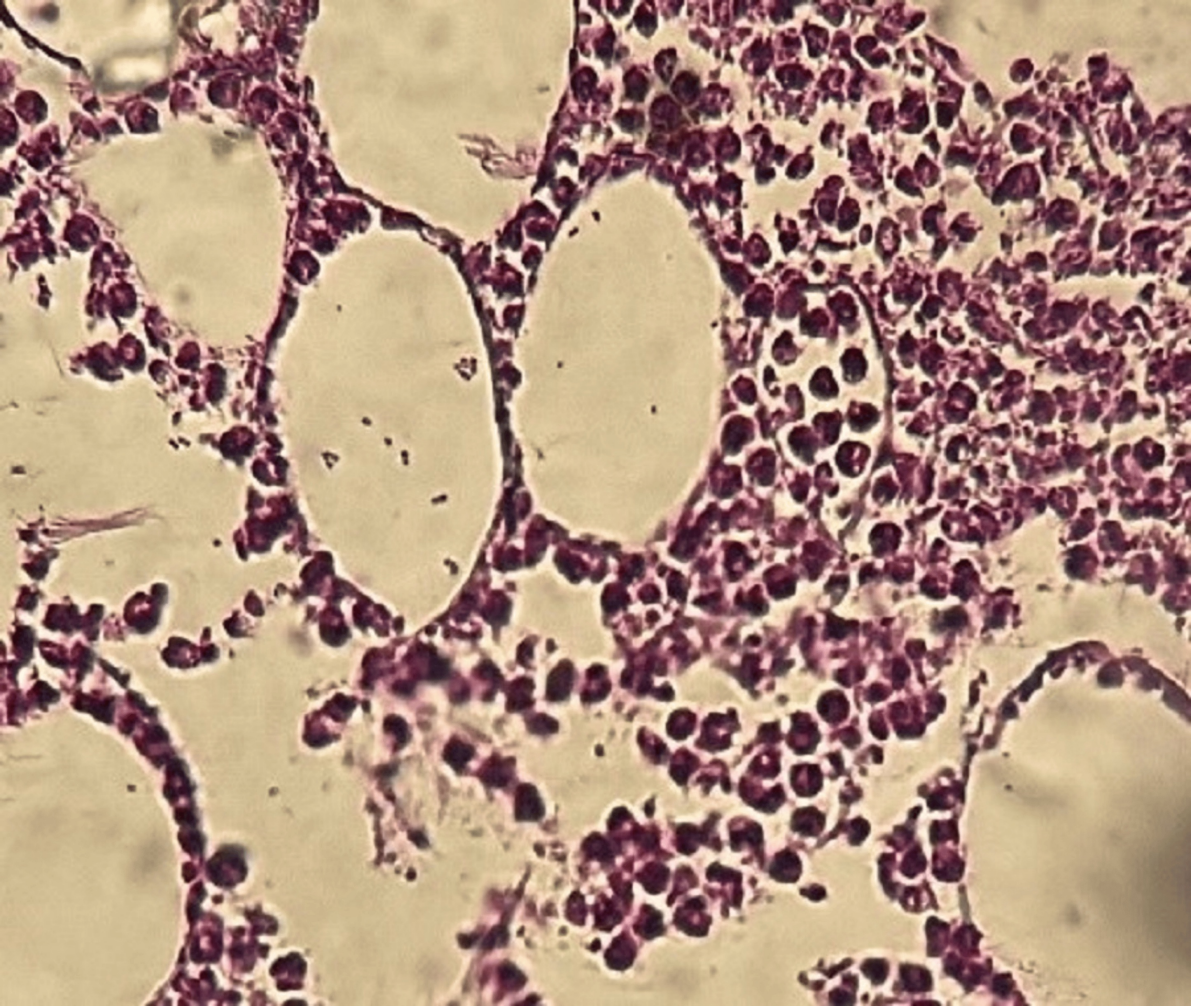
Histological image of the resected surgical specimen showing tumor proliferation with dual adipocytic and hematopoietic components (HE X 40)

The pathological examination of the surgical specimen revealed a well-defined, encapsulated rounded mass measuring 12 x 9 x 6 cm and weighing 380 g. The cut surface was brownish in appearance with scattered yellowish areas, extensively altered by hemorrhage, and firm and friable in consistency. Histological examination revealed mature adipose tissue mixed with hematopoietic elements. The diagnosis of an AML was then made (Figure 5).

The diagnostic error in the histological examination of the biopsy was explained by the fact that CD99 staining can be positive in several cell types, including endothelial cells, hepatocytes, ependymal cells, immature thymic T cells, and activated B lymphocytes. Indeed, our myelolipoma case included immature B lymphocytes, except that in order to confirm the diagnosis of Ewing's sarcoma, there must be intense membrane staining, which was lacking in the first histological reading, which revealed very weak staining (Figure 3). 

## Discussion

AMLs are benign, non-functioning tumors composed of mature adipose tissue and hematopoietic elements, first described by Gierke in 1905 [3]. Historically considered rare, AMLs are now increasingly identified due to the widespread availability of imaging techniques such as ultrasound, CT, and MRI, often performed for unrelated clinical indications [3]. Most AMLs remain small, usually under 5 cm, and are asymptomatic [3].

Giant AMLs, defined as lesions larger than 10 cm, represent an exceptional subset. A recent review covering the period 1981-2023 identified only approximately 15 cases reported in the literature [2]. These tumors typically present in middle-aged patients, with an average age around 50 years and a slight female predominance (ratio ~1.5) [2]. Most lesions are unilateral, and the left adrenal gland appears slightly more frequently involved [4]. In the PGIMER Chandigarh series of 15 cases, the mean tumor diameter was approximately 14 cm (± 6 cm), with the largest reaching 26 cm [4]. Despite their impressive size, giant AMLs have no documented metastatic potential and remain benign [2].

Large AMLs, however, are more often symptomatic. Patients may present with abdominal or flank pain, palpable masses, abdominal distension, or symptoms related to compression of adjacent organs such as the kidney or major vessels [4]. Systemic manifestations such as anemia or fever have also been described [4]. Rarely, rapid enlargement may lead to spontaneous hemorrhage or rupture [5]. The characteristics of the present case, marked tumor volume, heterogeneous imaging features, and symptomatic presentation, align closely with the typical clinical profile of a giant AML.

The diagnostic challenge of giant AMLs arises from their radiologic heterogeneity. Although the presence of macroscopic fat is suggestive, the coexistence of dense soft-tissue components, hemorrhage, necrosis, or calcifications can mimic malignant adrenal or retroperitoneal tumors, including adrenocortical carcinoma, retroperitoneal liposarcoma, lymphoma, adenoma, or metastatic disease [2]. Several cases reported in the literature required surgical excision before the diagnosis could be confirmed histopathologically, despite suggestive imaging features [6,7]. Furthermore, although the fatty component is typical of an AML, heterogeneity (fatty areas + higher densities, possible necrosis, hemorrhages, calcifications) complicates radiological interpretation [7,8]. The combined use of ultrasound, CT, and MRI is essential to improve diagnostic accuracy, yet histopathology remains the definitive standard [3,6].

Management depends primarily on the tumor size, symptoms, and diagnostic certainty. Small, asymptomatic AMLs (<5-6 cm) are generally managed with radiologic surveillance [3]. In contrast, large or symptomatic lesions, or those in which malignancy cannot be reliably excluded, should be surgically resected [4,5]. For giant AMLs (>10 cm), adrenalectomy remains the treatment of choice [2]. Although minimally invasive approaches are increasingly used for adrenal lesions, their role in very large, adherent, or anatomically complex tumors remains debated. Several authors have advocated open surgery to reduce intraoperative complications and ensure complete resection [6,9]. Surgical management is therefore both diagnostic and therapeutic, confirming the benign nature of the tumor while relieving symptoms caused by mass effect [7].

The etiopathogenesis of AMLs is not fully understood. Proposed mechanisms include metaplastic transformation of reticuloendothelial cells within the adrenal microvasculature in response to chronic stimuli such as necrosis, infections, stress, or prolonged ACTH stimulation, as well as activation of ectopic adrenal hematopoietic elements [2,10]. Some studies have suggested associations with obesity, hypertension, and diabetes, possibly reflecting chronic adrenal stimulation, although these relationships remain speculative and unproven [9].

The present case of a large, heterogeneous giant AML illustrates many of the key challenges described in the literature. It demonstrates the potential for these lesions to mimic malignant tumors radiologically, emphasizes the need for comprehensive clinical and imaging evaluation, and reinforces the importance of histopathological confirmation when doubt persists. In settings where large retroperitoneal tumors are frequently assumed to be malignant, this case underscores the importance of personalized, risk-adapted decision-making. By contributing to the limited number of reported cases, it enhances clinical awareness and strengthens the understanding of this rare but distinctive entity.

## Conclusions

A giant AML remains a rare clinical entity, whose large size and radiological heterogeneity can easily suggest a malignant tumor, particularly an adrenocortical carcinoma or retroperitoneal liposarcoma. This case is a prime example of the diagnostic pitfall posed by this benign tumor when its size greatly exceeds the usual dimensions and its imaging is atypical. The management of such masses requires a rigorous multidisciplinary assessment, incorporating clinical, biological, and radiological analysis and, where necessary, histopathological confirmation. In situations where doubts persist or in the presence of compressive symptoms, surgical resection remains the treatment of choice, allowing both diagnostic uncertainty to be resolved and the mass effect to be addressed. This observation highlights the importance of maintaining a high level of suspicion while considering benign diagnoses, even in the face of impressive radiological features. It also serves as a reminder that, despite their sometimes-pseudo-malignant appearance, AMLs have an excellent prognosis once diagnosed and treated appropriately. Through this case, we highlight the need to continue documenting and sharing these atypical presentations in order to better understand the morphological spectrum of AMLs, refine diagnostic criteria, and optimize therapeutic strategies for these rare but potentially misleading tumors.
